# Pediatric Allergy to Poultry Meat: A Case of Egg-Bird Syndrome

**DOI:** 10.7759/cureus.102766

**Published:** 2026-02-01

**Authors:** Marta I Dias, Rita S Pereira, Paulo R Santos, Tânia Monteiro, Marisa Carvalho, Marcia Quaresma

**Affiliations:** 1 Pediatrics, Unidade Local de Saúde de Trás-os-Montes e Alto Douro, Vila Real, PRT; 2 Pediatrics and Neonatology, Unidade Local de Saúde de Trás-os-Montes e Alto Douro, Vila Real, PRT

**Keywords:** alpha-livetin (gal d 5), cross-reactivity, egg-bird syndrome, food allergy, omalizumab, poultry meat allergy

## Abstract

Poultry meat allergy is a rare condition that can present as a primary allergy or a secondary syndrome, such as the egg-bird syndrome. This syndrome involves primary sensitization to egg yolk (alpha-livetin (Gal d 5)) leading to cross-reactivity with poultry meat. We describe the case of a 12-year-old male patient who presented with a history of primary egg allergy and allergy to dog epithelium. He subsequently developed an allergy to poultry meat. Immunoallergological evaluation showed sensitization to egg yolk, chicken meat, and alpha-livetin (Gal d 5). Sodium dodecyl sulfate-polyacrylamide gel electrophoresis (SDS-PAGE) immunoblotting confirmed immunoglobulin E (IgE) reactivity to alpha-livetin and avian muscle serum albumin, with no cross-reactivity to dog epithelium. This confirmed the diagnosis of egg-bird syndrome. Due to severe asthma and multiple food allergies impacting his quality of life, the patient was treated with omalizumab. This case highlights the clinical complexity of the egg-bird syndrome and the utility of molecular diagnostics in differentiating cross-reactivities. Omalizumab proved effective in managing the patient's polysensitization and asthma, improving quality of life.

## Introduction

Poultry meat allergy is a rare condition affecting children and adults, with an unknown prevalence due to limited epidemiological data [1]. This allergy manifests in two distinct forms: primary or secondary poultry meat allergies.

Primary poultry meat allergy results from direct sensitization to heat-stable meat allergens through intestinal exposure, unrelated to egg allergy [2]. It typically presents in adolescents and young adults, though symptoms may begin earlier in childhood. The allergenic molecules involved in genuine poultry meat allergy are not well known, but studies have shown alpha-parvalbumin and protein myosin light chain 1 as relevant allergens [2].

Secondary poultry meat allergy arises from cross-reactivity through two mechanisms, bird-egg or egg-bird syndrome. Both syndromes are caused by sensitization to serum albumin, specifically alpha-livetin (Gal d 5). Because this protein is present in both egg yolk and poultry meat, it serves as the molecular basis for this cross-reactivity [3].

In bird-egg syndrome, sensitization occurs through respiratory exposure to bird feathers or droppings, followed by reactions to poultry meat and egg yolk. Initial cases were reported in adults with pet bird exposure. In contrast, egg-bird syndrome is more common in children, where primary sensitization occurs through intestinal exposure to egg yolk, leading to subsequent reactions to poultry meat and potential respiratory symptoms upon bird exposure [3]. We present a case of secondary poultry meat allergy in a 12-year-old male patient, illustrating the complexity of cross-reactive sensitization patterns in some pediatric patients.

## Case presentation

We present a 12-year-old male patient with a family history of asthma and crustacean allergy. He had outdoor dogs at home until age six but was currently living without pets. His allergic history began at age two, with immunoglobulin E (IgE)-mediated cutaneous reactions to undercooked eggs (before that age, he ate eggs without problem), followed by urticaria upon dog exposure at age three. By age six, he developed cutaneous and gastrointestinal symptoms to cashew. At age eight, he experienced pharyngeal pruritus upon poultry meat ingestion. By age nine, he exhibited allergic rhinitis and asthma triggered by dog contact.

Laboratory evaluation included total IgE and specific IgE levels, as well as molecular component testing (Table 1).

**Table 1:** Laboratory results. IgE: immunoglobulin E; IU/mL: international units per milliliter; kU/L: kilounits of allergen-specific IgE per liter; ISU-E: immuno solid-phase allergen chip (ISAC) standardized units (semi-quantitative measure for molecular spreading)

Component (unit)	Observed values	Reference range
Total IgE (IU/mL)	1380	<60
Specific IgE (kU/L)		
Egg yolk	12.3	<0.35
Egg white	5.8	<0.35
Cashew	4.08	<0.35
Chicken meat	3.46	<0.35
Molecular components (ISU-E)		
Ana o 3	3.7	<0.3
Cor a 9	1.0	<0.3
Gal d 5	4.2	<0.3
Gal d 3	2.0	<0.3
Can f 1	>100	<0.3
Can f 2	77	<0.3
Can f 4	100	<0.3
Can f 5	23	<0.3
Can f 6	87	<0.3
Fel d 1	1.6	<0.3
Fel d 4	1.6	<0.3

**Figure 1:**
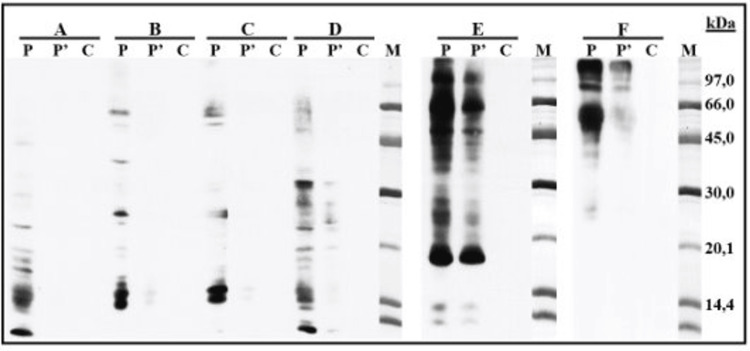
SDS-PAGE immunoblotting detecting IgE reactivity to poultry meat, dog epithelium, and alpha-livetin (Gal d 5). SDS-PAGE immunoblotting using the patient's serum at two dilutions (P, P’) and negative control serum (C). Protein extracts tested in each panel: cooked turkey breast (A), raw chicken breast (B), raw chicken thigh (C), cooked turkey thigh (D), dog epithelium extract (E), and purified alpha-livetin (Gal d 5) (F). Distinct IgE reactivity is observed against several poultry proteins, especially a prominent band corresponding to alpha-livetin (~66 kDa) in Lane F. IgE: immunoglobulin E; Lane M: molecular weight marker; SDS-PAGE: sodium dodecyl sulfate-polyacrylamide gel electrophoresis

**Figure 2:**
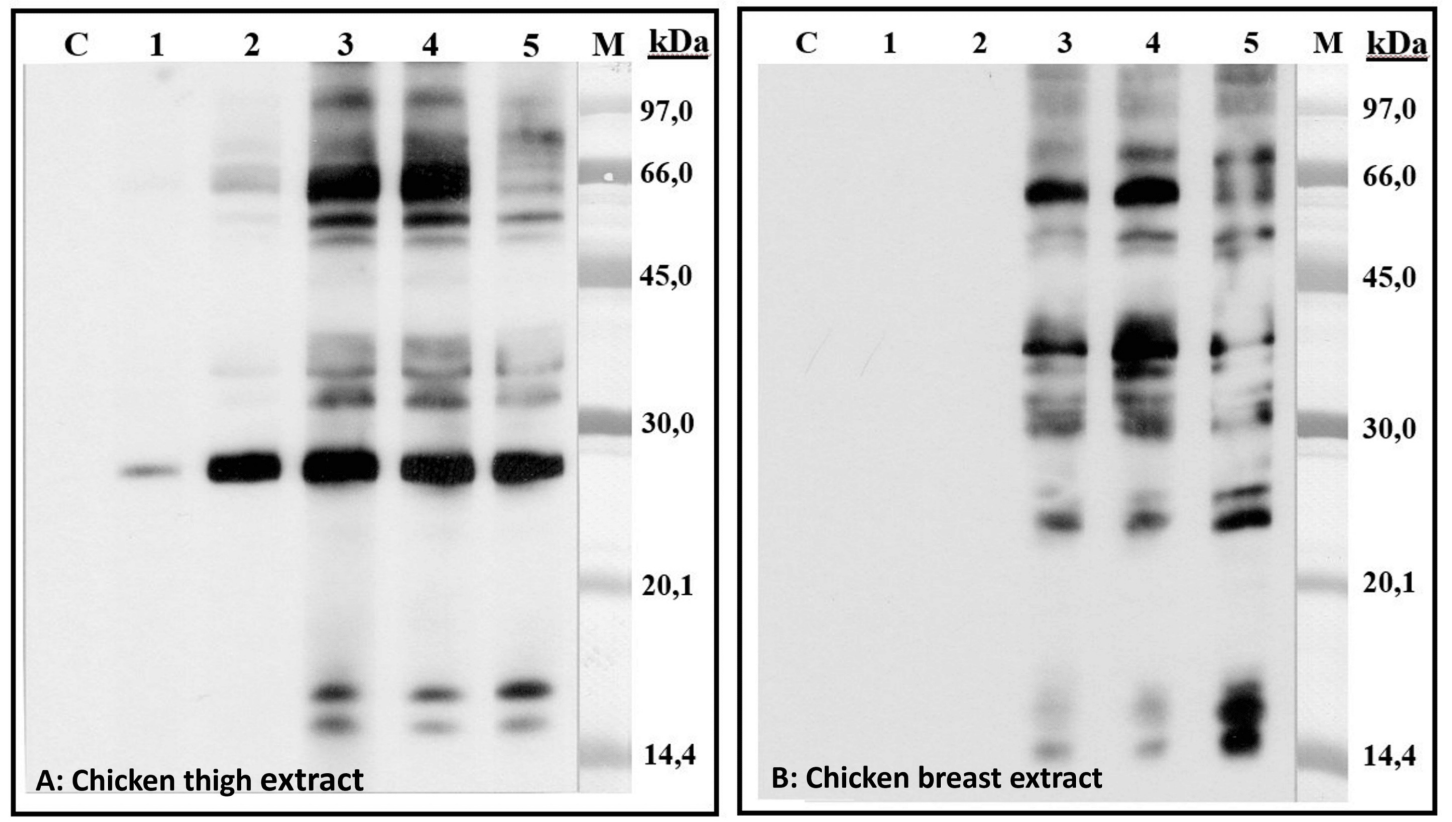
SDS-PAGE immunoblot inhibition assay assessing IgE cross-reactivity. Immunoblotting showing IgE reactivity against chicken thigh extract (panel A) and chicken breast extract (panel B). Patient serum was preincubated with various protein inhibitors before application to the strips. Lanes 1-5: patient serum preincubated with chicken thigh extract (Lane 1), chicken breast extract (Lane 2), dog epithelium extract (Lane 3), sunflower pollen extract (Lane 4), and purified alpha-livetin (Gal d 5) (Lane 5). Lane C: negative control serum from non-atopic individuals. Inhibition by chicken extracts and alpha-livetin markedly reduces IgE binding in both panels. No inhibition is observed with dog epithelium or sunflower pollen, indicating no cross-reactivity. IgE: immunoglobulin E; Lane M: molecular weight marker; SDS-PAGE: sodium dodecyl sulfate-polyacrylamide gel electrophoresis

To differentiate secondary poultry meat allergy due to hen's egg sensitization from cross-reactivity with dog serum albumin, sodium dodecyl sulfate-polyacrylamide gel electrophoresis (SDS-PAGE) immunoblotting was performed. This detected IgE reacting to chicken and turkey proteins, IgE reacting to alpha-livetin, and also IgE reacting to dog epithelial proteins (Figure 1). No cross-reactivity between poultry and dog proteins was found (Figure 2), supporting a diagnosis of secondary poultry meat allergy mediated by initial sensitization to Gal d 5 and subsequent cross-reactivity with avian muscle serum albumin.

The patient maintained an elimination diet (without pistachio, cashew, poultry, and undercooked egg). By age 10, omalizumab was initiated due to persistent asthma exacerbated by direct and indirect contact with dog epithelium in this patient with a complex food allergy. After 12 months, a better quality of life was achieved because reduced asthma exacerbations and an improvement in dietary restrictions were achieved.

## Discussion

This case illustrates the rare egg-bird syndrome, in which primary sensitization to Gal d 5 leads to cross-reactive allergy to poultry meat. The patient's history, described by cutaneous reactions to undercooked egg followed by pharyngeal pruritus to poultry meat ingestion, aligns with the classic presentation of this syndrome, where intestinal exposure to egg allergen triggers IgE-mediated responses to chicken muscle serum albumin [3].

The absence of cross-reactivity between chicken and dog serum albumin on immunoblotting is consistent with the literature, which shows low sequence homology between avian and mammalian serum albumins (sequence identity <30%) [4,5]. Gal d 5, a heat-labile allergen, explains the patient's tolerance to well-cooked egg but reactions to raw or undercooked forms. Its presence in egg yolk, poultry meat, and bird feathers/droppings creates a sensitization triad [3,5].

This case report describes a rare and complex food allergy profile, highlighting an egg-bird syndrome in which primary sensitization to alpha-livetin in egg yolk leads to secondary poultry meat allergy through cross-reactivity with avian muscle albumin. ImmunoCAP testing is crucial for distinguishing bird-egg syndrome from other cross-reactivities.

Omalizumab should be considered for polysensitized patients with severe food allergies and asthma, especially when dietary restrictions impair quality of life [6,7]. Future studies should explore epitope-specific immunotherapies to modulate Gal d 5-driven responses and investigate epitope mapping as a prognostic tool [5].

## Conclusions

This case highlights the diagnostic complexity of the rare egg-bird syndrome, particularly in differentiating it from the bird-egg syndrome and genuine poultry meat allergy. We demonstrated that primary sensitization to the heat-labile allergen alpha-livetin (Gal d 5) via the gastrointestinal tract can lead to secondary poultry meat allergy, necessitating a careful evaluation of patient history and specific molecular sensitization profiles. Furthermore, this report emphasizes the importance of a thorough immunological evaluation, including immunoblotting, to rule out cross-reactivity with dog serum albumin in patients with concomitant dog allergy.

Finally, the successful use of omalizumab in this case supports its consideration as a therapeutic strategy for polysensitized pediatric patients with severe asthma and complex food allergies, offering a significant improvement in quality of life.
